# Estimating the within‐subject (CV_I_) and between‐subject (CV_G_) biological variation of serum tryptase

**DOI:** 10.1002/iid3.578

**Published:** 2021-12-13

**Authors:** Birthe R. Skarbø, Erik W. Vinnes, Tore Wentzel‐Larsen, Marit S. Sylte, Torunn O. Apelseth

**Affiliations:** ^1^ Department of Medical Biochemistry and Pharmacology Haukeland University Hospital Bergen Norway; ^2^ Centre for Clinical Research Haukeland University Hospital Bergen Norway; ^3^ Centre for Child and Adolescent Mental Health The Regional Centres for Child and Adolescent Mental Health and Child Welfare, Eastern and Southern Norway Oslo Norway; ^4^ Norwegian Centre for Violence and Traumatic Stress Studies NORCE research Oslo Norway; ^5^ Department of Immunology and Transfusion Medicine Haukeland University Hospital Bergen Norway; ^6^ Department of War Surgery and Emergency Medicine Norwegian Armed Forces Medical Services Sessvollmoen Norway; ^7^ Faculty of Medicine University of Bergen Bergen Norway

**Keywords:** biological variation, reference change value, tryptase

## Abstract

**Background:**

Tryptase is used as a biomarker to support the diagnosis of anaphylaxis and hematologic diseases. In the event of a mast cell activation during anaphylaxis, a temporary increase in the concentration of tryptase may be seen. On the basis of clinical studies, an increase of 2 µg/L + 20% from basis level has been proposed as significant. To evaluate the increase in tryptase levels, the within‐subject (CV_I_) and between‐subject (CV_G_) biological variations should be known. This study was conducted to estimate the biological variation of tryptase and to identify the reference change value (RCV).

**Methods:**

Blood samples were collected from healthy volunteers once a week consecutively over a 10‐week period. Tryptase was measured by the use of a fluoroenzyme immunoassay (ImmunoCAP^TM^; Thermo Fisher Scientific), and linear mixed‐effects models were used to calculate the biological variation and RCV for both nontransformed and log‐transformed tryptase.

**Results:**

Fourteen presumably healthy young adults (six males and eight females, age 23–35 years) were included. The CV_I_ was 5.6% and the CV_G_ was 31.5% (nontransformed data). Log‐transformed data showed similar results. The analytical variation (CV_A_) was 6.3% and the RCV was 23.5%.

**Conclusions:**

Young healthy adults without ongoing allergic reactions show low within‐subject biological variation. Higher biological variation was observed between subjects.

## Graphical abstract

To evaluate an increase in tryptase levels in anaphylaxis, the within‐subject (CV_I_) and between‐subject (CV_G_) biological variations should be known. This study was conducted to estimate the biological variation of tryptase and to identify the reference change value.

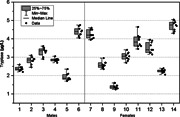

**Abbreviations**
CI: confidence interval
CV: coefficient of variation
CV_G_
: between‐subject biological variation
CV_I_
: within‐subject biological variation
EQAS: external quality assurance assessment scheme
QC: quality control
RCV: reference change value
SD: standard deviation

## 1 INTRODUCTION

Tryptase is a protease enzyme mainly stored in and released from mast cells.^1^, ^2^ In the event of an anaphylactic reaction, tryptase increases during the acute phase (1–4 h after the onset of symptoms), before returning to base levels approximately 12 h after the onset of the reaction. Therefore, serum tryptase is measured in both an acute sample and a sample taken either before or after the reaction. An international consensus equation has been proposed, defining an increase of at least 2 µg/L + 20% from the patient's baseline value to be considered a significant increase.^3^, ^4^ This equation has been evaluated in several clinical studies.^4^, ^5^, ^6^ It is, however, important for the clinician to be cognizant of the fact that anaphylaxis remains a clinical diagnosis.^7^, ^8^, ^9^ The measurement of tryptase is generally recommended for evaluation of the mechanism of anaphylaxis and remains the current gold standard biomarker.^6^, ^7^, ^10^, ^11^

Serum tryptase is also routinely utilized in the diagnosis of several nonanaphylactic situations. For instance, it is included as a minor criterion for the diagnosis of systemic mastocytosis according to WHO classification.^12^, ^13^ Tryptase has also shown potential as a biomarker in other myeloid diseases such as myeloproliferative neoplasms, myelodysplastic syndrome, and myeloid leukemia.^14^

Information on biological variation is important for the identification of pathological changes in laboratory parameters. No previous publications have systematically investigated the biological variation of tryptase, and it is not included in the EFLM Biological Variation Database.^15^ On the basis of clinical studies, an increase of 2 µg/L + 20% from basis level has been proposed as significant. To evaluate the increase in tryptase levels, the biological variations should be known. Therefore, the aim of this study is to estimate the within‐subject (CV_I_) and between‐subject (CV_G_) biologic variation and the reference change value (RCV) of tryptase.

The study was inspired by the EFLM recommendations in the checklist of biological variation studies.^16^, ^17^ The dataset was analyzed by linear mixed‐effects models suitable for data with repeated measures or other types of clustering.^18^

## 2 MATERIALS AND METHODS

### 2.1 Study design and ethics

The study was designed as a prospective observational study and was performed at the Department of Medical Biochemistry and Pharmacology at Haukeland University Hospital, Bergen, Norway. All study participants provided written informed consent before inclusion, and the study was approved by the Regional Committee for Medical and Health Research Ethics, Western Norway (REC Id.: 2017/1179). Data collection commenced on October 16, 2017 and was completed on December 18, 2017. Funding was provided by the Department of Medical Biochemistry and Pharmacology, Haukeland University Hospital.

### 2.2 Study population

All 14 recruited study participants were volunteers and presumed healthy laboratory staff. Inclusion criteria required participants to self‐report being in a *state of well‐being, feeling healthy*, and to be ≥18 years of age at the time of inclusion. Exclusion criteria required volunteers not to have any allergic reactions, surgeries, or blood transfusions 4 weeks before the commencement of the study.

### 2.3 Data collection

All participants completed a questionnaire about self‐reported allergies, exercise before the time of sampling, and the use of medication or prescription drugs. The utilized questionnaire is provided in Supporting Information.

### 2.4 Sample collection and handling

Blood samples were collected by standard phlebotomy once a week for 10 consecutive weeks, from October 16, 2017 to December 18, 2017. Phlebotomy was performed on the same day of the week (±1 day) and at the same time of the day (08:00–10:00 a.m.).

Blood samples were collected into 3.5 ml plastic serum‐separation Vacutainer gel tubes (Becton Dickson), and centrifuged for 10 min at 2000*g* in a swing‐out centrifuge at 20°C within 2 h after a clotting time of 30 min. All serum samples were transferred into four aliquotes (nunc‐tubes 0.5 ml) and placed in a −80°C freezer within 4 h after the time of phlebotomy. The stability of tryptase over several freeze‐thawing cycles has been shown not to be diminished up to four freeze‐thawing cycles and over 15 months.^19^

The analysis of tryptase was performed in two batches. The first batch was analyzed during the spring of 2018 and consisted of the samples from the first five participants. The second batch was analyzed in the fall of 2018 and consisted of the samples from the remaining nine participants. All serum samples were thawed 1 day before the time of analysis. Samples from the same participant were analyzed within the same run, and all tryptase measurements were performed using the same reagent lot. Finally, all samples were analyzed in duplicates (replicates) to estimate the analytical variation.

### 2.5 Assays

Analysis of tryptase was performed on Phadia 1000, ImmunoDiagnostics, (Thermo Fisher Scientific). The Phadia 1000 platform utilizes ImmunoCAPTM sandwich immunoassay with fluorescence detection. The manufacturer's reagents, calibrators, and internal quality (QC) control materials were utilized. The instrument performance was validated with internal QC samples at two levels (high and low) before each set of analyses. The laboratory also participates in external quality assessment schemes (EQAS), in which tryptase is included (UK NEQAS—tryptase). Ideal performance characteristics regarding the trueness of reported values by EQAS were achieved during both the spring and fall batches, and no drift of measurements was suspected between spring and fall. The limit of quantitation (LOQ) for tryptase analyzed on Phadia 1000 is 1.0 µg/L.^20^

### 2.6 Data and statistical analysis

Statistical analysis of data was performed by use of linear mixed‐effects models to account for a multilevel clustering.^18^ The model estimates both fixed and random effects, where the fixed effect represents the overall estimated mean while random effects include standard deviations (SDs) at the within‐subject, between‐subject, and analytical levels. Mixed‐effects models were estimated for both untransformed and log‐transformed tryptase. Estimates of coefficients of variation (CV) for CV_I_, CV_G,_ and analytical variation (CV_A_) levels were estimated in both models, together with the RCV.

For outlier analysis, we used the procedure described in Fraser and Harris (1989, Appendix, Section IV, Subsections A–C),^21^ both for tryptase and log‐transformed tryptase. First deviant differences between duplicates were identified based on duplicate differences using half the squared difference as a variance measure, and comparing the maximum divided by the sum with the critical value for a Cochran *c* test based on all these variances. Next, duplicate means are computed and their variance computed for each person. A test statistic is computed as the maximum variance divided by the sum, and compared with the critical value for a Cochran *c* test based on the number of persons and the mean number of observations per person. Finally, individual means are computed and test statistics are computed as the difference between the two extreme cases in each direction, divided by the total range of the means. Reed's criterion is used, checking whether any of these rations are above one‐third. If any outliers are identified, a sensitivity analysis is performed by re‐estimating the mixed‐effects models for tryptase and log‐transformed tryptase without the outliers, and the results are compared with the original analysis.

In the model for untransformed data of tryptase, CVs are estimated as SDs for random variation at each level, divided by the overall estimated mean, and multiplied by 100 for interpretation as a percentage. RCV, in percent, is estimated as $\sqrt{2}\times Z\times \sqrt{{\text{CV}}_{I}^{2}+{\text{CV}}_{A}^{2}}$, with a *Z* value of 1.96 (bidirectional, with a 95% probability).^21^ In the model for log‐transformed data of tryptase, CVs at each level are determined by the corresponding random effects as, for example, described by Fokkema et al.,^22^ $100\times \sqrt{\exp\left(\sigma^{2}\right)-1}$, where multiplication by 100 is for interpretation as a percentage, and σ is the SD for random variation at each level, or for a combination of levels. RCV, in percent, is estimated as described in.^22^ First, a deviation was computed for log‐transformed tryptase as $D=\sqrt{2}\times 1.96\times \sqrt{{\text{σ}}_{I}^{2}+{\text{σ}}_{A}^{2}}$, next separate RCVs were computed for changes *up* or *down* as 100×[exp(D)−1] for changes *up*, and 100×[exp(−D)−1] for changes *down*, respectively. Separate RCVs for changes up and down were necessary because a log‐normally distributed variable is asymmetric and necessarily positive.

Confidence intervals (CIs) for all estimates of CV and RCV were computed by percentiles based on parametric bootstrapping by 10 000 simulations from the estimated models. Since the distribution of tryptase in this data set is not known, CIs were computed with random draws from both the untransformed and the log‐transformed distributions, also for estimates based on the opposite model.

To check for a possible time trend, the models were repeated with the time variable (week number, from 1 to 10) as a covariate in the fixed effects part of the model. The models were also repeated with gender and age as covariates. The index of individuality was calculated by CV_I_/CV_G_.^21^

Data collection and statistical analysis were performed using SPSS (IBM SPSS Statistics 24) and R (version 3.4.1–4.0.4). SPSS was used for data collection including questionnaire answers and analytical results. R was used for all other statistical analyses, using the R package *nlme* for the mixed‐effects models.

## 3 RESULTS

**Figure 1:**
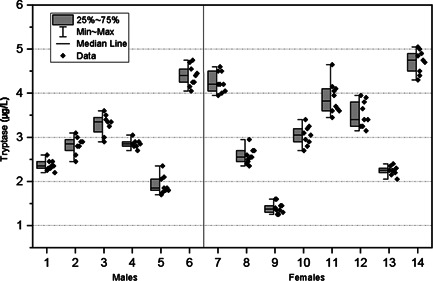
Biological variation of tryptase (µg/L) for each participant (*n* = 14). Participants 1–6 were male and 7–14 were female. Data points depict the mean values of the duplicates (*n* = 2) for each sample. The gray box represents the 25% and 75% percentiles, whiskers show range (min–max), and the median value is shown by the horizontal line

Fourteen volunteers were included in the study. The mean age of the participants was 25.8 years (range 23–35). Six of the participants were male (43%) and eight were female (57%). A total of 126 samples were included, yielding a mean number of nine samples per subject. No participant missed more than 2 weeks during the duration of the study and none of the duplicate runs were missing (two replicates per sample). The mean tryptase concentration was 3.1 µg/L (95% CI, 2.6–3.6). The range was 1.2–5.4 µg/L with a median value of 3.0 µg/L. No significant difference in tryptase values between males and females (*p* ≥ .449), or significant relationships with age (*p* ≥ .113) were found. There was also no evidence for a time trend (*p* ≥ .377). The results are shown in Figure 1, and the estimates from the mixed‐effects models are summarized in Table 1. The index of individuality was calculated to be 0.18. None of the measurement results were lower than the LOQ.

**Table 1:** Results from the linear mixed‐effects models for estimation of coefficients of variation (CV, %) of biological and analytical variation, including 95% confidence intervals (CIs)

	Based on the model for nontransformed tryptase	Based on the model for log‐transformed tryptase				
	Estimate	CI, simulated tryptase	CI, simulated log tryptase	Estimate	CI, simulated log tryptase	CI, simulated tryptase
Between‐subject CV, %	31.5	18.8–45.4	20.9–50.6	35.2	21.5–49.8	19.5–81.2
Within‐subject CV, %	5.6	4.1–7.4	3.8–7.4	5.4	3.9–6.6	4.3–15.4
Analytical CV, %	6.3	5.2–7.8	5.9–8.2	6.6	5.8–7.4	5.7–17.3
RCV, %	23.5	19.5–28.8	22.0–28.4	–	–	–
RCV, %, down	–	–	–	−21.0	−22.9 to −19.2	−47.6 to −19.4
RCV, %, up	–	–	–	26.6	23.8–29.6	24.1–90.7

Abbreviation: RCV, reference change value.

In the first step of the outlier analysis, one outlier is identified for tryptase duplicates, one person had duplicates 4.0 and 5.4 in Week 10. For log‐transformed tryptase, another outlier was identified, another person had duplicates 1.9 and 2.8 in Week 6. The mean number of weeks per person was 9 (range 8–10), and since this was an appreciably more common number the critical values in the second step were based on 9 weeks. With 14 persons the critical value for the Cochran *c* test was 0.192. The test statistic was .190 for tryptase and .143 for log‐transformed tryptase. Thus, no outlier was identified. In the third step, the two extreme differences divided by total range were 0.16 and 0.10 for tryptase, and 0.26 and 0.06 for log‐transformed tryptase, well below 1/3.

In the sensitivity analysis, the mixed effects were repeated without the outlying observations for one person in Week 9 and the other person in Week 6. The results are shown in Table 2.

**Table 2:** Results from linear mixed‐effects models for estimation of coefficients of variation (%) of biological and analytical variation with and without outlier exclusion, including 95% confidence intervals (CIs)

	Original		Sensitivity	
Tryptase	Estimate	95% CI	Estimate	95% CI
Mean	3.09	2.57, 3.61	3.09	2.57, 3.61
Standard deviation between persons	0.98	0.63, 1.51	0.98	0.68, 1.41
Standard deviation within persons	0.17	0.14, 0.22	0.19	0.15, 0.23
Standard deviation within duplicates	0.20	0.17, 0.22	0.17	0.15, 0.19
Log‐transformed tryptase				
Mean	1.074	0.893, 1.256	1.073	0.890, 1.255
Standard deviation between persons	0.342	0.233, 0.503	0.345	0.234, 0.507
Standard deviation within persons	0.054	0.042, 0.069	0.055	0.044, 0.069
Standard deviation within duplicates	0.066	0.058, 0.075	0.059	0.052, 0.067

## 4 DISCUSSION

Multiple studies have investigated baseline levels of tryptase in healthy children and adults^23^, ^24^, ^25^, ^26^; however, we have not been able to identify any publications systematically estimating the biological variation over a time period with consecutive measurements. Brown et al.^27^ conducted a controlled insect venom challenge (*n* = 64) in which tryptase was measured on all participants 14 weeks before the challenge. In the group who did not experience any symptoms (*n* = 53), tryptase did not differ more than 2 µg/L from the sample 14 weeks before the study, compared to the prechallenge sample taken at the commencement of the provocation. The study however compares only two separate measurements 14 weeks apart and thus cannot provide a dataset to perform estimates of CV_I_ and CV_G_. Their maximum difference of 2 µg/L might, however, be in concordance with our findings of the maximum difference in tryptase of 1.4 µg/L for a participant during the 10 weeks.

The calculated CV_A_ found in our study is higher than the minimum analytical performance specification (0.25 × CV_I_) as described by Fraser,^21^ meaning that CV_A_ may cause an increased (>25%) variability between test results. This poses some diagnostic limitations, as larger changes in tryptase are required to confirm significant changes between test results.

Our findings confer an index of individuality for tryptase of 0.18 (CV_I_/CV_G_). By convention an index <0.6 is considered *markedly individual*, favoring the reporting of individual results rather than by population‐based reference intervals.^21^, ^28^ Thus, our findings support the use of consecutive measurements of tryptase to assess mast cell activation in the event of anaphylaxis.

Using the mean value of our data (3.1 µg/L), the calculated RCV represents a change of 0.73 µg/L. For RCV based on log‐transformed tryptase, the similar changes are 0.65 µg/L down and 0.82 µg/L up. This indicates that significant changes may be detected earlier by RCV than by using the proposed 2 µg/L + 20% from the basis level. However, it must be noted that our data set is based on young healthy adults and, therefore, may not be applicable to an older population as it has been shown that tryptase levels significantly increase with age.^24^, ^25^

The dataset used in the present article represents multiple measurements on a limited number of persons and is, therefore, not a good basis for the investigation of distributional assumptions. Since the distribution of tryptase in this dataset is not known, estimates were computed both assuming a normal and a log‐normal distribution. The observed differences in CVs and RCVs between models for untransformed and log‐transformed tryptase were, however, minimal. A few confidence limits were elevated, particularly the highest confidence limits for the absolute values. This may be due to data being generated from a normal distribution and modeled with a log‐normal distribution. Therefore, it may be better to avoid the log transformation if the distribution of tryptase in the population under study is not known to be skewed.

The CV_G_ was considerably higher than the CV_I_ and CV_A_. This finding is in accordance with studies of reference intervals of tryptase in healthy individuals showing a wide range of 1–15 µg/L, while asymptomatic individuals maintain the same level of tryptase over time.^29^

Generally, there were small differences between original analyses and analyses excluding the two identified outlying pairs of duplicates.

The main weakness of our study is the skewed demographics towards a young age of the participants (mean = 25.8, range 23–35 years). Therefore, in future studies, it would be advantageous to include an older population. Another weakness of our study is that the samples were not all analyzed on the same day. This may have contributed to a higher CV_G_. We consider it a strength to have calculated CV from both nontransformed and log‐transformed data, as the distribution of tryptase in our study population is unknown. Another strength is that most participants followed the measurement regime consecutively for the duration of 10 weeks. In addition, no duplicate analysis was missed allowing us to satisfactory calculate the CV_A_.

## 5 CONCLUSION

In this study, we have observed low CV_I_ for tryptase in healthy adults without ongoing allergic reactions. Higher biological variation was observed between subjects. Our findings indicate that significant changes in tryptase levels may be detected earlier by using the RCV values than by the proposed 2 µg/L + 20%.

## CONFLICT OF INTERESTS

The authors declare that there are no conflict of interests.

## AUTHOR CONTRIBUTIONS

Birthe R. Skarbø coordinated the study, performed phlebotomy of the study participants, and prepared samples for analysis. Birthe R. Skarbø and Erik W. Vinnes wrote the manuscript. Tore Wentzel‐Larsen performed the statistical analysis. Figures/tables were created by Erik W. Vinnes and Tore Wentzel‐Larsen. Torunn O. Apelseth and Marit S. Sylte designed the study and provided guidance as senior scientists. All authors have approved and provided revisions to the final draft of the manuscript.

## Supporting information

Supporting information.

## Data Availability

The data that support the findings of this study are available from the corresponding author upon reasonable request.
