# Supplementary material for: Estimating the within‐subject (CVI) and between‐subject (CVG) biological variation of serum tryptase
Source: Immun Inflamm Dis. 2021 Dec 13;10(4):e578. doi: 10.1002/iid3.578 (PMC8959422; doi:10.1002/iid3.578)
Supplement: Supplementary file 1 — Supporting information. [file IID3-10-e578-s001.pdf]

R. script BV tryptase, article ID: IID3578

```
library(foreign)
library(nlme)

ba <- read.spss("database - biologisk variasjon alle.sav", to.data.frame=TRUE)
summary(ba)
# de aktuelle variablene
bb <- ba[,c("Id","ukenr","Tryptase","Tryptase2","Alder","KjÃ¸nn","Panel")]
bb
# sorterer pÃ¥ Id og ukenr, antakelig allerede gjort
bb <- bb[order(bb$Id,bb$ukenr),]
summary(bb)
# en missing pÃ¥ kjÃ¸nn og alder
# ser pÃ¥ missingene pÃ¥ tryptase
table(is.na(bb$Tryptase), is.na(bb$Tryptase2), useNA="always") # de samme
# missingene pÃ¥ de to tryptase-mÃ¥lingene, tar vekk de som har missing pÃ¥ begge
bb <- bb[is.na(bb$Tryptase)==0|is.na(bb$Tryptase2)==0,]
summary(bb) # da er begge tryptasemÃ¥lingene gyldige for alle,
# og ikke misisng pÃ¥ kjÃ¸nn eller alder da
bb
summary(by(bb$ukenr, bb$Id, min)) # alle Id har ukenr 1 fÃ¸rst
summary(by(bb$ukenr, bb$Id, max)) # alle Id har ukenr 9 eller 10 sist
# sjekker konsistens av kjÃ¸nn og alder
for (ii in unique(bb$Id)) {
  tabk <- table(bb$KjÃ¸nn[bb$Id==ii])
  taba <- table(bb$Alder[bb$Id==ii])
  tabk <- tabk[tabk>0]
  taba <- taba[taba>0]
  if (length(tabk)!=1) print(tabk)
  if (length(taba)!=1) print(taba)
```

```
} # end sjekk konsistens i kjÃ¸nn og alder, ok
# kjÃ¸nn og alder
summary(bb[bb$ukenr==1,c("KjÃ¸nn","Alder")])
sort(bb$Alder[bb$ukenr==1])# stemmer med tabell 1 og det fÃ¸r den

# oppsett med ei linje for hver observasjon, som grupperte data
# med to klyngenivÃ¥er, uker innen id og duplikater innen uke
nbb <- dim(bb)[1]
b2 <- data.frame(id=rep(bb$Id,2), uke=rep(bb$ukenr,2),
            dupl=c(rep(1,nbb),rep(2,nbb)),
            kj=rep(bb$KjÃ¸nn,2), alder=rep(bb$Alder,2),
            tryp=c(bb$Tryptase, bb$Tryptase2))
b2 <- b2[order(b2$id,b2$uke,b2$dupl),]
b2 <- groupedData(b2, formula=tryp~uke|id/uke)
b2

# stikkprÃ¸vesjekk
sel <- sample(unique(bb$Id),1)
bb[bb$Id %in% sel,c("Id","ukenr","Alder","KjÃ¸nn","Tryptase","Tryptase2")]
b2[b2$id %in% sel,]

# modell for tryptase
lmetr <- lme(tryp~1, random=~1, data=b2)
anova(lmetr, type="marginal")
summary(lmetr)
intervals(lmetr)

# Results under figuren
summary(lmetr) # samlet 126 prÃ¸ver stemmer
ba[,c("Id","ukenr","Tryptase", "Tryptase2")]
# dette fra artiklen stemmer ikke
```

```
# and none of the duplicate runs were missing (2 replicates per sample).
# det er alltid slik at begge mÃ¥linger finnes eller mangler samme uke
# det stemmer at ingen har mer enn to uker med missing tryptase
nuker.id <- as.numeric(by(b2$id, b2$id, length))/2 # /2 virker i og med
# at fÃ¸rste mÃ¥ling og duplikatet er gyldige samtidig
names(nuker.id) <- 1:14
nuker.id
mean(nuker.id) # gjennomsnitt 9 stemmer, men hadde ikke trengt Ã¥ vÃ¦re et heltall
mean(b2$tryp) # stemmer med artiklen
sd(b2$tryp) # stemmer ikke med artiklen



# grafisk modellsjekk
plot(lmetr) # litt Ã¸kende tendens, kan skyldes skeiv tryptase
qqnorm(lmetr)# noen fÃ¥ outliere i begge retninger, ellers ok

# CV
sdrand.lmetr <- c(as.numeric(intervals(lmetr)$reStruct$id[2]),
          as.numeric(intervals(lmetr)$reStruct$uke[2]),
          as.numeric(intervals(lmetr)$sigma[2]))
names(sdrand.lmetr) <- c("inter","intra","analyt")
sdrand.lmetr
fixed.mean <- as.numeric(intervals(lmetr)$fixed[2])
fixed.mean
cvrand.lmetr <- 100*sdrand.lmetr/fixed.mean
cvrand.lmetr # noen mindre avvik fra tabell 2 i artiklen
# kombinert intra og analytisk
cvrand.lmetr.intra.an <- sqrt(cvrand.lmetr["intra"]^2 + cvrand.lmetr["analyt"]^2)
cvrand.lmetr.intra.an

# RCV
```

```
rcv.lmetr <- sqrt(2)*qnorm(.975)*sqrt(cvrand.lmetr["intra"]^2 + cvrand.lmetr["analyt"]^2)
rcv.lmetr # 23.5%, greit

# modell for log tryptase
lmeltr <- lme(I(log(tryp))~1, random=~1, data=b2)
anova(lmeltr, type="marginal")
summary(lmeltr)
intervals(lmeltr)

# grafisk modellsjekk
plot(lmeltr) # en stor outlier oppover, ellers stort sett ok,
# ingen klar vifteform
qqnorm(lmeltr)# en stor outlier oppover, ellers stort sett ok

# identifiserer avvikende observasjoner
b2.res <- data.frame(b2, trres=resid(lmetr, type="p"), ltrres=resid(lmeltr, type="p"))
b2.res[abs(b2.res$trres)>2|abs(b2.res$ltrres)>2,] # det vesentlige her er innen
# id 9 uke 10 og id 11 uke 6
b2.res[b2.res$id %in% c(9,11),] # id 9 uke 10 litt hÃ¸y tryptase sÃ¦rlig andre mÃ¥ling,
# id 11 uke 6 vanskelig Ã¥ si hva som slÃ¥r ut. gjÃ¸r ikke noe med dette

# CV for tryptase ut fra modell pÃ¥ logskala, beregning som for Fokkema
sdrand.lmeltr <- c(as.numeric(intervals(lmeltr)$reStruct$id[2]),
        as.numeric(intervals(lmeltr)$reStruct$uke[2]),
        as.numeric(intervals(lmeltr)$sigma[2]))
names(sdrand.lmeltr) <- c("inter","intra","analyt")
sdrand.lmeltr
# kombinert standardavvik for logtransformert tryptase, intra og analyt
sdrand.lmetr.intra.an <- sqrt(sdrand.lmeltr["intra"]^2 + sdrand.lmeltr["analyt"]^2)
sdrand.lmetr.intra.an
# tilsvarende CV, full oppdeling, i prosent
```

```
cv.lmeltr <- 100*sqrt(exp(sdrand.lmeltr^2)-1)

cv.lmeltr.intra.analyt <- 100*sqrt(exp(sdrand.lmeltr["intra"]^2 + sdrand.lmeltr["analyt"]^2)-1)

cv.lmeltr.intra.analyt

# sammenlikning med CV ut fra utransformert tryptase

data.frame(utransformert=cvrand.lmetr, transformert=cv.lmeltr)

# CV i prosent for samlet intra og analytisk variasjon

# det likner, med logtransformert tryptase er det litt mindre

# prosentvis CV intra og litt mer inter og analyt


# RCV i prosent for samlet intra og analytisk variasjon

# (Fokkema side 1602, nederst i andre og Ã¸verst i tredje spalte)

rcv.lmeltr.intra.an.grunnlag <- sqrt(2)*qnorm(.975)*sqrt(sdrand.lmeltr["intra"]^2 +
sdrand.lmeltr["analyt"]^2)

rcv.lmeltr.intra.an <- c(100*(exp(-rcv.lmeltr.intra.an.grunnlag) - 1),
100*(exp(rcv.lmeltr.intra.an.grunnlag) - 1))

names(rcv.lmeltr.intra.an) <- c ("down","up")

rcv.lmeltr.intra.an

# sammenlikning med utransformert

rcv.lmetr # godt mellom grensene ned og opp for logtransformert tryptase




# oppsett for simulering/parametrisk bootstrapping

summary(b2)

intervals(lmetr)

intervals(lmeltr)

trlme.simf <- function(grdata=b2, lmeobj=lmetr, lmelobj=lmeltr, nsim=10000, check=0) {

  ngrd <- dim(grdata)[1]

  nidgr <- length(unique(grdata$id))

  nidukegr <- length(unique(100*grdata$id + grdata$uke))

  n.pr.id <- as.numeric(by(grdata$id, grdata$id, length))

  n.pr.iduke <- as.numeric(by(100*grdata$id + grdata$uke,
```

```
                        100*grdata$id + grdata$uke, length))
fixmean <- as.numeric(intervals(lmeobj)$fixed[2])
fixlmean <- as.numeric(intervals(lmelobj)$fixed[2])
randsd <- c(as.numeric(intervals(lmeobj)$reStruct$id[2]),
          as.numeric(intervals(lmeobj)$reStruct$uke[2]),
          as.numeric(intervals(lmeobj)$sigma[2]))
randlsd <- c(as.numeric(intervals(lmelobj)$reStruct$id[2]),
          as.numeric(intervals(lmelobj)$reStruct$uke[2]),
          as.numeric(intervals(lmelobj)$sigma[2]))
grdata$trypexp <- fixmean
grdata$tryplexp <- fixlmean
grdata$trypsim <- fixmean
grdata$typlsim <- fixlmean
grdata$randinter <- NA
grdata$randintra <- NA
grdata$randanalyt <- NA
simres <- data.frame(intercv=rep(NA,nsim), intracv=rep(NA,nsim),analytcv=rep(NA,nsim),
                  rcv=rep(NA,nsim),
                  intercvl=rep(NA,nsim), intracvl=rep(NA,nsim),analytcvl=rep(NA,nsim),
                  rcvl=rep(NA,nsim),
                  lintercv=rep(NA,nsim), lintracv=rep(NA,nsim),lanalytcv=rep(NA,nsim),
                  lrcvd=rep(NA,nsim),lrcvu=rep(NA,nsim),
                  lintercvl=rep(NA,nsim), lintracvl=rep(NA,nsim),lanalytcvl=rep(NA,nsim),
                  lrcvdl=rep(NA,nsim), lrcvul=rep(NA,nsim),
                  nnpos=rep(NA,nsim))
for (sim in 1:nsim) {
  grdata$randinter <- rep(rnorm(n=nidgr), n.pr.id)
  grdata$randintra <- rep(rnorm(n=nidukegr), n.pr.iduke)
  grdata$randanalyt <- rnorm(n=ngrd)
  grdata$trypsim <- grdata$trypexp + randsd[1]*grdata$randinter +
    randsd[2]*grdata$randintra + randsd[3]*grdata$randanalyt
```

```
  grdata$tryplsim <- grdata$tryplexp + randlsd[1]*grdata$randinter +
    randlsd[2]*grdata$randintra + randlsd[3]*grdata$randanalyt
  if (check==1) {
    print(randsd)
    print(randlsd)
    print(grdata)
  }
  lmesim <- try(lme(trypsim~1, random=~1, data=grdata))
  lmesiml <- try(lme(I(exp(tryplsim))~1, random=~1, data=grdata))
  lmelsim <- try(lme(I(log(trypsim))~1, random=~1, data=grdata[grdata$trypsim>0,]))
  lmelsiml <- try(lme(tryplsim~1, random=~1, data=grdata))
  simres$nnpos[sim] <- sum(grdata$trypsim<=0)
  if (class(lmesim)=="lme") {
    intsim <- try(intervals(lmesim))
    if (class(intsim)=="intervals.lme") {
      if (check==1) print(intsim)
      sdrand.lmesim <- c(as.numeric(intsim$reStruct$id[2]),
                         as.numeric(intsim$reStruct$uke[2]),
                         as.numeric(intsim$sigma[2]))
      lmesim.mean <- as.numeric(intsim$fixed[2])
      if (lmesim.mean!=0) {
        simres[sim,c("intercv","intracv","analytcv")] <- 100*sdrand.lmesim/lmesim.mean
        simres[sim,"rcv"] <- sqrt(2)*qnorm(.975)*sqrt(simres[sim,"intracv"]^2 +
simres[sim,"analytcv"]^2)
      }
    } # end if intervals(lmesim) ok
  } # end if lmesim ok
  if (class(lmesiml)=="lme") {
    intsiml <- try(intervals(lmesiml))
    if (class(intsiml)=="intervals.lme") {
      if (check==1) print(intsiml)
```

```
      sdrand.lmesiml <- c(as.numeric(intsiml$reStruct$id[2]),
                 as.numeric(intsiml$reStruct$uke[2]),
                 as.numeric(intsiml$sigma[2]))
      lmesiml.mean <- as.numeric(intsiml$fixed[2])
      if (lmesiml.mean!=0) {
       simres[sim,c("intercvl","intracvl","analytcvl")] <- 100*sdrand.lmesiml/lmesiml.mean
       simres[sim,"rcvl"] <- sqrt(2)*qnorm(.975)*sqrt(simres[sim,"intracvl"]^2 +
simres[sim,"analytcvl"]^2)
      }
     } # end if intervals(lmesiml) ok
    } # end if lmesiml ok
    if (class(lmelsim)=="lme") {
     intlsim <- try(intervals(lmelsim))
     if (class(intlsim)=="intervals.lme") {
      if (check==1) print(intlsim)
      sdrand.lmelsim <- c(as.numeric(intlsim$reStruct$id[2]),
                 as.numeric(intlsim$reStruct$uke[2]),
                 as.numeric(intlsim$sigma[2]))
      simres[sim,c("lintercv","lintracv","lanalytcv")] <- 100*sqrt(exp(sdrand.lmelsim^2)-1)
      lgrunnlag <- sqrt(2)*qnorm(.975)*sqrt(sdrand.lmelsim[2]^2 + sdrand.lmelsim[3]^2)
      simres[sim,c("lrcvd","lrcvu")] <- c(100*(exp(-lgrunnlag) - 1), 100*(exp(lgrunnlag) - 1))
     } # end if intervals(lmelsim) ok
    } # end if lmelsim ok
    if (class(lmelsiml)=="lme") {
     intlsiml <- try(intervals(lmelsiml))
     if (class(intlsiml)=="intervals.lme") {
      if (check==1) print(intlsiml)
      sdrand.lmelsiml <- c(as.numeric(intlsiml$reStruct$id[2]),
                 as.numeric(intlsiml$reStruct$uke[2]),
                 as.numeric(intlsiml$sigma[2]))
      simres[sim,c("lintercvl","lintracvl","lanalytcvl")] <- 100*sqrt(exp(sdrand.lmelsiml^2)-1)
```

```
      lgrunnlagl <- sqrt(2)*qnorm(.975)*sqrt(sdrand.lmelsiml[2]^2 + sdrand.lmelsiml[3]^2)
      simres[sim,c("lrcvdl","lrcvul")] <- c(100*(exp(-lgrunnlagl) - 1), 100*(exp(lgrunnlagl) - 1))
     } # end if intervals(lmelsiml) ok
    } # end if lmelsiml ok
    if (sim==floor(sim/100)*100) print(sim)
  } # end for sim
  return(simres)
} # end function trlme.simf
# sjekk
trlme.simf(nsim=2, check=1)
# kjÃ¸ring
fÃ¸r <- Sys.time()
trlme.sim <- trlme.simf()
etter <- Sys.time()
fÃ¸r
etter
etter-fÃ¸r # snaut 32 minutter
summary(trlme.sim) # 50 sammenbrudd pÃ¥ lmelsim <- try(lme(I(log(trypsim))~1, random=~1,
# data=grdata[grdata$trypsim>0,])) #  generert uten logtrans, modellert med
# ett sammenbrudd med  lmelsiml <- try(lme(tryplsim~1, random=~1, data=grdata)) # ,
# generert med logtrans, modellert uten
# ingen sammenbrudd ved samsvar mellom modellering og simulering
table(trlme.sim$nnpos, useNA="always") # 9491 simuleringer uten dette, 1-32 av 252
# linjer med negativ generert tryptase ved generering uten logtrans

# konfidensintervaller
confprobs <- c(.025,.975)
# beregning
# CV inter
confint.trlme.sim.intercv <- quantile(trlme.sim$intercv, probs=confprobs, na.rm=TRUE) # gen orig,
mod orig
```

confint.trlme.sim.intercvl <- quantile(trlme.sim$intercvl, probs=confprobs, na.rm=TRUE) # gen log, mod orig

confint.trlme.sim.lintercv <- quantile(trlme.sim$lintercv, probs=confprobs, na.rm=TRUE) # gen orig, mod log

confint.trlme.sim.lintercvl <- quantile(trlme.sim$lintercvl, probs=confprobs, na.rm=TRUE) # gen log, mod log

CV intra

confint.trlme.sim.intracv <- quantile(trlme.sim$intracv, probs=confprobs, na.rm=TRUE) # gen orig, mod orig

confint.trlme.sim.intracvl <- quantile(trlme.sim$intracvl, probs=confprobs, na.rm=TRUE) # gen log, mod orig

confint.trlme.sim.lintracv <- quantile(trlme.sim$lintracv, probs=confprobs, na.rm=TRUE) # gen orig, mod log

confint.trlme.sim.lintracvl <- quantile(trlme.sim$lintracvl, probs=confprobs, na.rm=TRUE) # gen log, mod log

CV analyt

confint.trlme.sim.analytcv <- quantile(trlme.sim$analytcv, probs=confprobs, na.rm=TRUE) # gen orig, mod orig

confint.trlme.sim.analytcvl <- quantile(trlme.sim$analytcvl, probs=confprobs, na.rm=TRUE) # gen log, mod orig

confint.trlme.sim.lanalytcv <- quantile(trlme.sim$lanalytcv, probs=confprobs, na.rm=TRUE) # gen orig, mod log

confint.trlme.sim.lanalytcvl <- quantile(trlme.sim$lanalytcvl, probs=confprobs, na.rm=TRUE) # gen log, mod log

RCV

confint.trlme.sim.rcv <- quantile(trlme.sim$rcv, probs=confprobs, na.rm=TRUE) # gen orig, mod orig

confint.trlme.sim.rcvl <- quantile(trlme.sim$rcvl, probs=confprobs, na.rm=TRUE) # gen log, mod orig

confint.trlme.sim.lrcvd <- quantile(trlme.sim$lrcvd, probs=confprobs, na.rm=TRUE) # gen orig, mod log, down

confint.trlme.sim.lrcvu <- quantile(trlme.sim$lrcvu, probs=confprobs, na.rm=TRUE) # gen orig, mod log, up

confint.trlme.sim.lrcvdl <- quantile(trlme.sim$lrcvdl, probs=confprobs, na.rm=TRUE) # gen log, mod log, down

confint.trlme.sim.lrcvul <- quantile(trlme.sim$lrcvul, probs=confprobs, na.rm=TRUE) # gen log, mod log, down

```
# resultater, sammenfatting
# CV inter
cvrand.lmetr[1] # orig
confint.trlme.sim.intercv   # gen orig, mod orig
confint.trlme.sim.intercvl  # gen log, mod orig
cv.lmeltr[1] # log
confint.trlme.sim.lintercv  # gen orig, mod log
confint.trlme.sim.lintercvl # gen log, mod log
# CV intra
cvrand.lmetr[2] # orig
confint.trlme.sim.intracv   # gen orig, mod orig
confint.trlme.sim.intracvl  # gen log, mod orig
cv.lmeltr[2] # log
confint.trlme.sim.lintracv  # gen orig, mod log
confint.trlme.sim.lintracvl # gen log, mod log
# CV analyt
cvrand.lmetr[3] # orig
confint.trlme.sim.analytcv   # gen orig, mod orig
confint.trlme.sim.analytcvl  # gen log, mod orig
cv.lmeltr[3] # log
confint.trlme.sim.lanalytcv  # gen orig, mod log
confint.trlme.sim.lanalytcvl # gen log, mod log
# rcv
rcv.lmetr # orig
confint.trlme.sim.rcv    # gen orig, mod orig
confint.trlme.sim.rcvl   # gen log, mod orig
rcv.lmeltr.intra.an[1] # log, down
confint.trlme.sim.lrcvd  # gen orig, mod log, down
confint.trlme.sim.lrcvdl # gen log, mod log, down
rcv.lmeltr.intra.an[2] # log, up
confint.trlme.sim.lrcvu  # gen orig, mod log, up
```

```
confint.trlme.sim.lrcvul # gen log, mod log, down


# index of indiciduality, ii=CV_I/CV_G, CV within/CV between,
# CV intra/CV inter
cvrand.lmetr[2]/cvrand.lmetr[1] # orig
cv.lmeltr[2]/# log



# sjekk av artikkel Manuskript_160321.doc
# Abstract, Results
summary(bb)
length(unique(bb$Id)) # antallet 14 ok
length(unique(bb$Id[bb$KjÃ¸nn=="Mann"]))   # 6 menn stemmer
length(unique(bb$Id[bb$KjÃ¸nn=="Kvinne"])) # 8 kvinner stemmer
summary(bb$Alder) # 23-35 Ã¥r stemmer
summary(lmetr)$tTable
intervals(lmetr)
# sjekk Results om estimated mean, ok
summary(b2$tryp) # sjekk range i Results, ok

# Discussion, stÃ¸rste forskjell 1,2
rangedifff <- function(xx) {
  len <- length(xx)
  rangex <- range(xx, na.rm=TRUE)
  rdx <- rangex[2] - rangex[1]
  return(rep(rdx,len))
} # end function rangedifff
b2.rangediff.tryp <- unlist(by(b2$tryp, b2$id, rangedifff))
summary(b2.rangediff.tryp)
data.frame(id=b2$id, uke=b2$uke, tryp=b2$tryp, trd=b2.rangediff.tryp)
data.frame(id=b2$id, uke=b2$uke, tryp=b2$tryp, trd=b2.rangediff.tryp)[b2.rangediff.tryp>1,]
```

```
# individualitetsindeks, CVA/CVI

# utransformert tryptase

c(cvrand.lmetr[2],cvrand.lmetr[1],cvrand.lmetr[2]/cvrand.lmetr[1])

# logtransformert tryptase

c(cv.lmeltr[2],cv.lmeltr[1],cv.lmeltr[2]/cv.lmeltr[1])



# omregning fra RCV tilbake til tryptase-skalaen (Discussion 0,73 Âµg/L)

# utransformert tryptase

c(rcv.lmetr, summary(lmetr)$tTable[1], (rcv.lmetr/100)*summary(lmetr)$tTable[1])

# 0,73 er ok

# logrtransformert tryptase, ned

c(rcv.lmeltr.intra.an[1], summary(lmetr)$tTable[1],
(rcv.lmeltr.intra.an[1]/100)*summary(lmetr)$tTable[1])

# logrtransformert tryptase, opp

c(rcv.lmeltr.intra.an[2], summary(lmetr)$tTable[1],
(rcv.lmeltr.intra.an[2]/100)*summary(lmetr)$tTable[1])



# Data- and statistical analysis, undersÃ¸kelse av tidstrend,

# gjentar modellene med tid som forklaringsvariabel

lmetr.uke <- lme(tryp~uke, random=~1, data=b2)

anova(lmetr.uke, type="marginal")

lmeltr.uke <- lme(I(log(tryp))~uke, random=~1, data=b2)

anova(lmeltr.uke, type="marginal")

# kjÃ¸nn og alder

lmetr.ka <- lme(tryp~kj+alder, random=~1, data=b2)

lmeltr.ka <- lme(I(log(tryp))~kj+alder, random=~1, data=b2)

anova(lmetr.ka, type="marginal")

anova(lmeltr.ka, type="marginal")
```

```
# Fraser og Harris, outliere
summary(b2)
# kopi for outlier-analyse (Fraser og Harris), heretter FH
b2fh <- b2[,c("id","uke","dupl","tryp")]
# sorterer pÃ¥ nytt for sikkerhets skyld
b2fh <- b2fh[order(b2fh$id,b2fh$uke,b2fh$dupl),]
# hjelpefunksjoner for Ã¥ lage hjelpevariabler for FH
firstf <- function(xx) c(1,rep(0,length(xx)-1))
lenrepf <- function(xx) rep(length(xx),length(xx))
fhvarduplf <- function(xx) rep(.5*(xx[2]-xx[1])^2, length(xx))
b2fh$firstindupl <- unlist(by(b2fh$id, 100*b2fh$id+b2fh$uke, firstf))
b2fh$ndupl <- unlist(by(b2fh$id, 100*b2fh$id+b2fh$uke, lenrepf))
table(b2fh$ndupl) # bare 2, ingen manglende duplikater
b2fh$fhvarindupltr <- unlist(by(b2fh$tryp, 100*b2fh$id+b2fh$uke, fhvarduplf))
# ser pÃ¥
summary(b2fh$fhvarindupltr[b2fh$firstindupl==1])
# testobservator
testa.tryp <- max(b2fh$fhvarindupltr[b2fh$firstindupl==1])/
  sum(b2fh$fhvarindupltr[b2fh$firstindupl==1])
testa.tryp
# kritisk verdi
krit.a <- 1/(1 + (126-1)/qf(1-.05/126,df1=1,df2=126-1))
krit.a # snaut 0,10
# flagger outliere for duplikatene for tryptase
b2fh$flagoutdupltryp <- as.numeric(b2fh$fhvarindupltr/
  sum(b2fh$fhvarindupltr[b2fh$firstindupl==1]) > krit.a)
table(b2fh$flagoutdupltryp[b2fh$firstindupl==1]) # en outlier
# idenfifisert, finner den
b2fh[b2fh$flagoutdupltryp==1,] # id 9, uke 10
# gjentar for log(tryptase)
b2fh$fhvarinduplltr <- unlist(by(log(b2fh$tryp), 100*b2fh$id+b2fh$uke, fhvarduplf))
```

```
testa.ltryp <- max(b2fh$fhvarinduplltr[b2fh$firstindupl==1])/
  sum(b2fh$fhvarinduplltr[b2fh$firstindupl==1])
testa.ltryp
b2fh$flagoutduplltryp <- as.numeric(b2fh$fhvarinduplltr/
                                    sum(b2fh$fhvarinduplltr[b2fh$firstindupl==1]) > krit.a)
table(b2fh$flagoutduplltryp[b2fh$firstindupl==1]) # en outlier
# idenfifisert, finner den
b2fh[b2fh$flagoutduplltryp==1,] # id 11, uke 6

# appendix B, gjennomsnitt i duplikatene og
# varianser av dem for hver person
gjrepf <- function(xx) rep(mean(xx), length(xx))
varrepf <- function(xx) rep(var(xx), length(xx))
b2fh$gjdupltryp <- unlist(by(b2fh$tryp, 100*b2fh$id+b2fh$uke, gjrepf))
b2fh$gjduplltryp <- unlist(by(log(b2fh$tryp), 100*b2fh$id+b2fh$uke, gjrepf))
b2fhb <- b2fh[b2fh$firstindupl==1,c("id","uke","gjdupltryp","gjduplltryp")]
b2fhb$firstinid <- unlist(by(b2fhb$uke, b2fhb$id, firstf))
b2fhb$nuke <- unlist(by(b2fhb$id, b2fhb$id, lenrepf))
b2fhb$vargjtryp <- unlist(by(b2fhb$gjdupltryp, b2fhb$id, varrepf))
b2fhb$vargjltryp <- unlist(by(b2fhb$gjduplltryp, b2fhb$id, varrepf))
b2fhbtaba2 <- b2fhb[b2fhb$firstinid==1,c("id","nuke","vargjtryp","vargjltryp")]
summary(b2fhbtaba2) # 8:10 uker, gjennomsnitt nÃ¦r 9
table(b2fhbtaba2$nuke) # 3 pÃ¥ 8, 8 pÃ¥ 9, 3 pÃ¥ 10, de aller fleste pÃ¥ 9
# testobservatorer trinn B
testobsb.tryp <- max(b2fhbtaba2$vargjtryp)/sum(b2fhbtaba2$vargjtryp)
testobsb.ltryp <- max(b2fhbtaba2$vargjltryp)/sum(b2fhbtaba2$vargjltryp)
testobsb.tryp
testobsb.ltryp
# kritisk verdi
kritb.nuke8 <- 1/(1 + (14-1)/qf(1-.05/14,df1=8-1,df2=(14-1)*(8-1)))
kritb.nuke9 <- 1/(1 + (14-1)/qf(1-.05/14,df1=9-1,df2=(14-1)*(9-1)))
```

```
kritb.nuke10 <- 1/(1 + (14-1)/qf(1-.05/14,df1=10-1,df2=(14-1)*(10-1)))
c(kritb.nuke8,kritb.nuke9,kritb.nuke10)
# begge testobservatorene, bÃ¥de for tryp og for log(tryp),
# er under kritisk verdi hvis 9 uker legges til grunn for
# beregning av kritisk verdi. Hvis 10 uker legges til grunn
# er det litt over for tryptase, ser etter
b2fhbtaba2$vargjtryp.sum <- b2fhbtaba2$vargjtryp/sum(b2fhbtaba2$vargjtryp)
b2fhbtaba2 # litt over kritisk grense med 10 uker/id, for id 14,
# klart under ellers, bare for tryp, ikke for log(tryp)
b2fhb$gjidtryp <- unlist(by(b2fhb$gjdupltryp, b2fhb$id, gjrepf))
b2fhb$gjidltryp <- unlist(by(b2fhb$gjdupl ltryp, b2fhb$id, gjrepf))
# trinn C, Reeds kriterium
# beregner person-gjennomsnitt, kan bygge pÃ¥ duplikat-gjennomsnittene
# i og med at det alltid er to duplikater
b2fhc <- b2fhb[b2fhb$firstinid==1, c("id","nuke","gjidtryp","gjidltryp")]
b2fhc
nid <- dim(b2fhc)[1] # 14
# omrÃ¥de og forskjell for person-gjennomsnittene i tryp og ltryp
range.gjidtryp <- range(b2fhc$gjidtryp)
range.gjidltryp <- range(b2fhc$gjidltryp)
rangediff.gjidtryp <- range.gjidtryp[2] - range.gjidtryp[1]
rangediff.gjidltryp <- range.gjidltryp[2] - range.gjidltryp[1]
# fÃ¸rst sortere pÃ¥ gjidtryp
b2fhc <- b2fhc[order(b2fhc$gjidtryp),]
b2fhc
# de to ekstremdifferansene
exdiffctryp <- c(b2fhc$gjidtryp[2]-b2fhc$gjidtryp[1],
        b2fhc$gjidtryp[nid]-b2fhc$gjidtryp[nid-1])
exdiffctryp
# som andel av rangediff
exdiffctryp/rangediff.gjidtryp # godt under 1/3 av rangeduff
```

```
# deretter sortere pÃ¥ gjidtryp
b2fhc <- b2fhc[order(b2fhc$gjidltryp),]
b2fhc
# de to ekstremdifferansene
exdiffcltryp <- c(b2fhc$gjidltryp[2]-b2fhc$gjidltryp[1],
        b2fhc$gjidltryp[nid]-b2fhc$gjidltryp[nid-1])
exdiffcltryp
# som andel av rangediff
exdiffcltryp/rangediff.gjidltryp
# stÃ¸rste ekstremdifgferanse som andel av rangediff er 0,26
# i hvert fall noe under 1/3

# mixed effects-modellene i hovedanalyse og uten de mulige outlierene
b2$outla <- as.numeric((b2$id==9&b2$uke==10)|(b2$id==11&b2$uke==6))
# sjekk
summary(b2$outla[!(b2$id %in% c(9,11))]) # ingen, ok
b2[b2$id %in% c(9,11),] # ok

# modell for tryptase uten outlierne
lmetru <- lme(tryp~1, random=~1, data=b2[b2$outla==0,])
lmeltru <- lme(I(log(tryp))~1, random=~1, data=b2[b2$outla==0,])
# utransformert tryptase
anova(lmetr, type="marginal")
anova(lmetr, type="marginal") # ikke stor forskjell
summary(lmetr)
summary(lmetru)
intervals(lmetr)
intervals(lmetru) # ikke stor forskjell
# logtransformert tryptase
anova(lmeltr, type="marginal")
anova(lmeltru, type="marginal") #
```

```
summary(lmeltr)

summary(lmeltru)

intervals(lmeltr)

intervals(lmeltru)
```
